# Risk Factor Analysis of Delayed Intracerebral Hemorrhage After Coil Embolization of Unruptured Cerebral Aneurysms

**DOI:** 10.3389/fneur.2020.584596

**Published:** 2020-10-30

**Authors:** Wonsoo Son, Dong-Hun Kang

**Affiliations:** ^1^Department of Neurosurgery, School of Medicine, Kyungpook National University, Daegu, South Korea; ^2^Department of Radiology, School of Medicine, Kyungpook National University, Daegu, South Korea

**Keywords:** antiplatelate activity, risk factor, intracranial aneurysm, embolization, cerebral hemorrhage

## Abstract

**Background:** We sought to analyze diffusion-weighted imaging (DWI) and dual antiplatelet therapy (DAPT) for risk factors of delayed intracerebral hemorrhage (d-ICH) after coil embolization for an unruptured intracranial aneurysm (UIA).

**Methods:** A total of 539 aneurysms were analyzed in this study. Ruptured and flow diverter cases were excluded. All aneurysms enrolled in this study were treated with stent-assisted or simple coiling techniques. Before the procedure, all patients administered (DAPT). After the procedure, patients who underwent stent-assisted coil embolization were given DAPT, and patients who underwent simple coiling were given single antiplatelet therapy (SAPT) only during their admission. The response of the antiplatelet agent was assessed the day before the procedure with The VerifyNow assay. DWI MRI and CT were obtained routinely the next day after the procedure. d-ICH was defined as an intracerebral hemorrhagic lesion identified in follow up CT at least 48 h after the procedure.

**Results:** A larger positive lesion on day 1 DWI MRI (*p* = 0.001), the value of PRU (*p* = 0.002), and the inhibition rate (*p* = 0.025) were considered meaningful risk factors for d-ICH in univariate analysis. Accordingly, larger DWI positivity (OR = 83.73, 95% CI = 11.132–712.886, *P* = 0.001) and PRU (OR = 0.98, 95% CI = 0.972–0.999, *P* = 0.033) reached statistical significance in multivariate analysis.

**Conclusions:** Thromboembolic infarction may work as an initiating factor, and antiplatelet medication may work as an aggravating factor. We might suggest that a tailored reduction in antiplatelet agents could help reduce d-ICH when a larger volume of post-procedural thromboembolic infarction is seen on 1-day follow-up DWI MRI.

## Introduction

Antiplatelet agents such as aspirin and clopidogrel are essential for preventing thromboembolic complications during endovascular procedures for unruptured cerebral aneurysms (UIA) (1–4). With more complex coil embolization, such as balloon- or stent-assisted coiling and flow diversion, the risk of thromboembolism is more likely to increase. Thus, dual antiplatelet therapy (DAPT) is generally recommended in those cases to avoid thromboembolic complications (2).

However, concern about bleeding complications may increase during such procedures when DAPT is administered (4–8). Among hemorrhagic complications, intraprocedural events are mostly related to aneurysm rupture, adjacent vessel dissection, or perforation, which are not theoretically associated with antiplatelet usage. But delayed intracerebral hemorrhage (d-ICH), which is detected not immediately but a few days after the procedure, might increase in incidence via antiplatelet usage. Although the incidence of d-ICH is quite low, once it occurs, it can result in severe morbidity and mortality (4, 9, 10).

There are several studies regarding d-ICH after endovascular treatment of an UIA, but most were linked to flow diverters (11–17). Based upon our experience, d-ICH can occur not only in cases with a flow diverter but also in coiling cases, as antiplatelet maintenance is necessary in both situations (especially when a stent is used in coiling cases). However, risk factor analysis about d-ICH confined to coil embolization has not been sufficiently investigated. Accordingly, we sought to determine the risk factors of d-ICH after coil embolization for UIA.

## Materials and Methods

### Patients

The retrospective studies involving human participants were reviewed and approved by the institutional review board at Kyoungpook National University Hospital. The patients provided their written informed consent to participate in this study. Data were analyzed from patients who underwent coil embolization for cerebral aneurysms at our institute from July 2014 to August 2019. During the given period, a total of 697 patients with 697 aneurysms (ruptured, *n* = 140; unruptured, *n* = 557) were treated with an endovascular procedure.

We excluded patients diagnosed with ruptured aneurysms and patients who underwent flow diverter treatment (*n* = 15). To distinguish from d-ICH, we also excluded hemorrhagic event cases (*n* = 3) that occurred during the procedure. After exclusion, 539 patients with 539 UIA were finally enrolled. Demographic and clinical data collected included age, sex, and several risk factors (hypertension, diabetes, dyslipidemia, smoking and alcohol history, history of previous stroke, and history of previous heart disease).

### Antiplatelets Dosing Protocol Before and After Coil Embolization

The antiplatelet regimen before coil embolization at our institute involved DAPT. Aspirin and clopidogrel were administered at doses of 100 and 75 mg for 5 days before the procedure. The response of DAPT was assessed the day before the procedure with the VerifyNow assay (Accumetrics, San Diego, California, USA). Each target value of clopidogrel inhibition and aspirin inhibition was under 220 PRU and 550 ARU, respectively. When a test result did not fall within the reference range, a booster administration of DAPT was done for each patient before the procedure. After the procedure, patients who underwent stent-assisted coil embolization were given DAPT, and patients who underwent simple coiling were given single aspirin therapy (SAPT) only during their admission. Patients who had unexpected events during the procedure, such as acute thrombosis, were given DAPT after the procedure, even when the stent did not deploy.

### Procedure

All procedures were performed by a highly experienced neurointerventionist. Under general anesthesia, an initial bolus of 50 IU/kg heparin was injected intravenously at the beginning of the procedure. The target value of activated clotting time (ACT) was at 2–2.5 times baseline throughout the procedure and was checked every 30 min. The booster heparin was injected at a dose of 1,000 units/h. All catheters were flushed by using a continuous irrigation system with heparinized saline to avoid embolism caused by an air bubble. All flushed saline was heparinized (1,000 IU/100 mL), and guiding catheters and microcatheters were exposed to a continuous heparinized drip.

Standard approaches were performed through the left common femoral artery. A 6-French guiding catheter was advanced to the internal carotid artery (in case of anterior circulation aneurysms) or the vertebral artery (in case of posterior circulation aneurysms). After placing a microcatheter, embolization coils were inserted into the aneurysm sac. A variety of different coils were used to fill the sac. All aneurysms analyzed in this study were treated with stent-assisted techniques or simple coiling techniques. When we use stents, the type of stents varied depending on the situations; however, the Neuroform EZ and Neuroform Atlas (Stryker Neurovascular, Kalamazoo, MI, USA) were most commonly used.

After completing the procedures, post-procedural angiography was performed to see if there was evidence of embolic events. After completing the procedure, all patients were observed for 24 h in the neurosurgical intensive care unit and were continued on a regimen of subcutaneous heparin injection. Upon discharge, antiplatelet medication was stopped for patients who did not require stent insertion. If a stent was inserted, the patient received DAPT with it lasting for at least 3–6 months. After that, the regimen was changed to single antiplatelet administration and was continued.

### Postoperative MRI and Identification of Delayed ICH

Diffusion-weighted imaging (DWI) MRI was obtained routinely on the next day after the procedure, usually about 24 h after. DWI positive lesions were measured manually on axial images in the institutional picture archiving and communication system (PACS, INFINITT Healthcare, South Korea). A positive sign on follow-up DWI was defined as a high signal intensity lesion regardless of the side of the coiled aneurysm. We divided them into a “Microembolic DWI positive group” and a “Larger DWI positive group.” Only an area more than 10 mm^2^ was counted as larger DWI positive.

A routine follow-up brain CT scan was performed two times after the procedure, immediately after the procedure, and at the outpatients' department after 1–2 weeks later. An additional CT scan was taken under the physician's discretion when a patient complained of aggravated headache or newly developed neurodeficits. d-ICH was defined as an intracerebral hemorrhagic lesion identified in follow up CT at least 48 h after the procedure. Most cases identified d-ICH via a CT scan at the outpatients' department. Among 539 patients, eight with post-procedural d-ICH were identified after the procedure. Accordingly, we divided the patients into two groups, a d-ICH positive group (*n* = 8) and a d-ICH negative group (*n* = 531). We compared several characteristics and risk factors between the two groups.

### Statistical Analysis

Statistical analysis was performed using SPSS 26.0 (SPSS Inc. Chicago, IL, United States). We performed Fisher's exact test for the analysis of normal variables. Mann-Whitney U tests were used for the analysis of numerical variables. Then we performed multivariate logistic regression analysis to identify the independent risk factors of delayed ICH. Statistical differences were considered significant at *P*-values < 0.05.

## Results

### Patient Characteristics

Among the 539 patients with an unruptured aneurysm that underwent coil embolization treatment, eight were identified as having delayed post-procedural d-ICH. The overall incidence of post-procedural d-ICH was 1.5%. Baseline characteristics of all patients are summarized in Table 1. There were more females in each group, but there were no baseline differences that reached statistical significance.

**Table 1:** Summary of baseline characteristics.

	**d-ICH positive (*n* = 8)**	**d-ICH negative (*n* = 531)**	***p*-value**
Gender (M/F)	3/5	106/425	0.207^a^
Age ± SD	55.13 ± 11.75	61.42 ± 10.92	0.107
Pre Antiplt med Hx	1 (12.5%)	100 (18.8%)	0.999
HTN	5 (62.5%)	243 (45.8%)	0.480
DM	0 (0%)	54 (10.2%)	0.999
Dyslipidemia	5 (62.5%)	138 (31.6%)	0.118
Pre stroke Hx	0 (0%)	67 (12.6%)	0.604
Pre heart Dz Hx	1 (12.5%)	35 (6.6%)	0.427
Smoking	1 (12.5%)	84 (15.8%)	0.999
Alcohol	2 (25.0%)	125 (23.5%)	0.999

None of the underlying risk factors reached statistical significance, such as previous antiplatelet medication (*p* = 0.999), previous stroke history (*p* = 0.604), previous heart disease history (*p* = 0.427), and the presence of several underlying diseases such as hypertension, diabetes, and dyslipidemia. Anatomical characteristics of the aneurysms also did not show significant differences between the two groups (*p* = 0.693) (Table 2). However, ICA aneurysms had the largest proportion in both groups.

**Table 2:** Summary of radiologic and anatomical characteristics of aneurysms.

	**d-ICH positive (*n* = 8)**	**d-ICH negative (*n* = 531)**	****p*-value***
Size of Ax (mm)	4.99 ± 0.75	6.28 ± 2.59	0.160
Location of Ax	ICA 5 (62.5%)	ICA 368 (69.3%)	0.693
	MCA 0 (0%)	MCA 31 (5.8%)	
	ACA 2 (25%)	ACA 73 (13.7%)	
	Posterior 1 (12.5%)	Posterior 59 (11.1%)	
Post procedure	7 (87.5%)	431 (81.2%)	0.362
DWI positive			
**Subgroup analysis of post op DWI lesions**			
No DWI lesion	1 (12.5%)	100 (18.8%)	0.001
Mircoembolic DWI lesion	0 (0%)	398 (75.0%)	
Larger DWI lesion	7 (87.5%)	33 (6.2%)	
Stent deployment	8 (100%)	350 (65.9%)	0.057
Type of stent	None 0 (0%)	None 181 (28.4%)	0.091
	Open cell 6 (75%)	Open cell 226 (48.2%)	
	Closed cell 2 (25%)	Closed cell 124 (23.4%)	

### DWI Findings 1-Day Post-procedure

The incidence of a positive sign on day 1 of post-procedure DWI was much higher in the ICH positive group (87.5%) than the negative group (6.2%) (Table 2). And this difference was statistically significant (*p* = 0.001). We divided patients more specifically in terms of DWI lesions. No DWI lesion was defined as definitely without a positive signal on DWI MRI. As described previously, we divided the “DWI positive” group into a “Microembolic DWI lesion” group and a “Larger DWI lesion” group. A microembolic DWI lesion is only composed of tiny dotted lesions on DWI MRI, where the number of lesions was not considered. If any of the lesions had a positive sign with an area of 10 mm^2^ or more, they were classified in the “Larger DWI lesion” group. Table 2 shows that the ICH positive group had a much higher portion of larger DWI lesions (87.5%) than in the ICH negative group (6.2%). In the ICH negative group, the majority were micro DWI lesions (75.0%, *p* = 0.001).

Stent deployment did not show a meaningful relationship in d-ICH occurrence in univariate analysis in Table 2. The *p*-value did not reach statistical significance, but it was marginal (*p* = 0.057).

### Response to Antiplatelet Medication

The values of VerifyNow and post-procedure antiplatelet regimen are summarized in Table 3. The value of the ARU showed no significant difference between the two groups in univariate analysis (*p* = 0.419). The value of ARU in the d-ICH positive group was 450.00 ± 44.48, which was lower than the value of the ICH negative group (446.38 ± 45.05). However, the differences in PRU values was statistically significant (*p* = 0.002). Further, the value of PRU in the ICH positive group was 154.01 ± 48.35, which was lower than the value for the ICH negative group (226.47 ± 67.09). The inhibition rate also showed statistical significance (*p* = 0.025). Its average value in the ICH positive group (35.50 ± 18.42) was higher than in the ICH negative group (19.10 ± 20.47). All patients in the d-ICH positive group and 65.3% of d-ICH negative group patients were given DAPT and showed no significant relationship in d-ICH occurrence.

**Table 3:** Summary of the response to antiplatelet medication of two groups.

	**d-ICH positive (*n* = 8)**	**d-ICH negative (*n* = 531)**	***p*-value**
ARU	446.38 ± 45.05	476.05 ± 66.59	0.419
PRU	154.01 ± 48.35	226.47 ± 67.09	0.002
Inhibition rate (%)	35.50 ± 18.42	19.10 ± 20.47	0.025
Post procedure	Dual 8 (100%)	Dual 400 (65.3%)	0.209
Antiplt medication	Single 0 (0%)	Single 131 (24.7%)	

### Clinical Data of the Eight Patients With d-ICH

**Figure 1:**
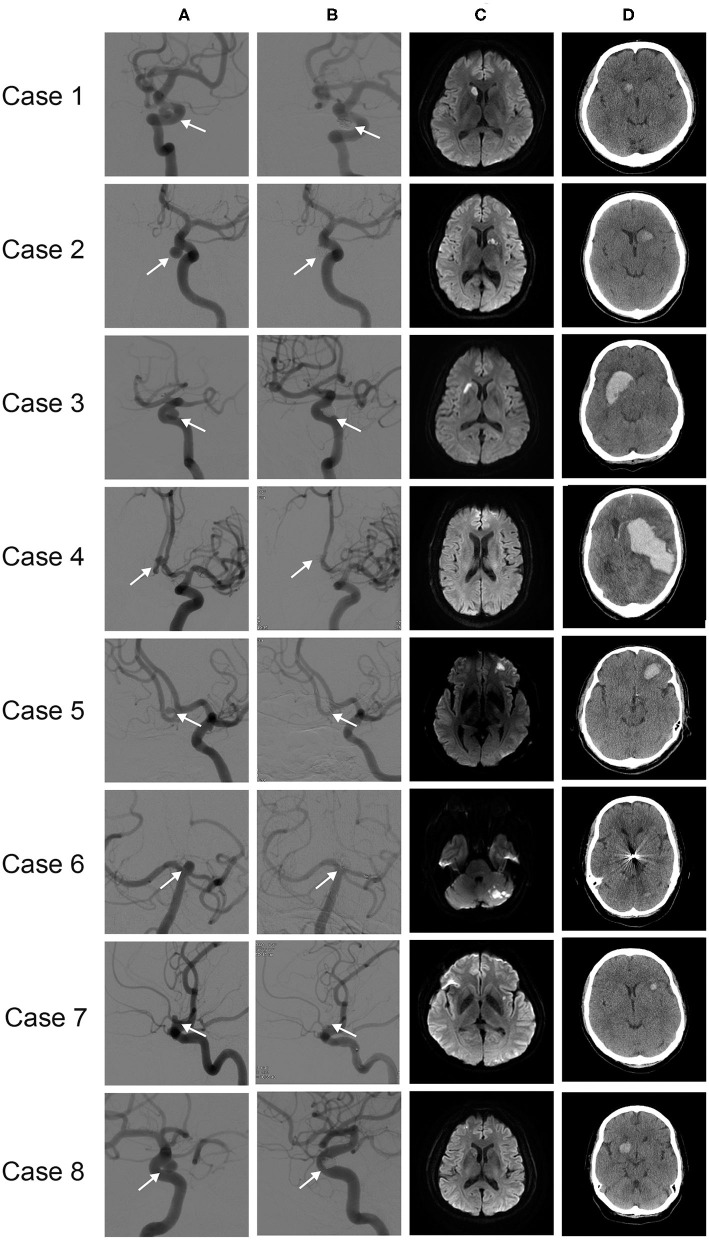
Eight cases of d-ICH: **(A)** pre-procedural angiograms, **(B)** post-procedural angiograms, with treated aneurysm indicated (arrow), **(C)** one-day post-procedural follow-up DWI, **(D)** follow-up CT scan, d-ICH identified.

Detailed clinical data of the eight patients with d-ICH are summarized in Table 4. Four images (pre-procedural DSA, post-procedural DSA, 1 day after post-procedure DWI, delayed CT scan) of each patient were demonstrated in Figure 1. When comparing DWI and CT scans, we found that d-ICH occurred exactly in the same location with the DWI positive lesion in seven of eight patients. Notably, all seven DWI positive lesions were in the larger DWI lesion subgroup. The location of each d-ICH varied differently, including one cerebellar lesion. The amount of hemorrhage ranged from 2 to 137 cc. The mean duration time from the procedure to the identification of d-ICH was 11.8 days.

**Table 4:** Clinical data of eight patients with d-ICH.

**Case No**	**Location of Ax**	**Size of Ax (mm)**	**ARU**	**PRU**	**Inhibition Rate (%)**	**DWI positivity, size subgroup and location**	**Location of ICH**	**Amount of ICH (cc)**	**Onset days of d-ICH from the procedure**
1	ICA dorsal, Rt	4.7	498	184	16	Yes, Larger (CN, Rt)	CN, Rt	2	11
2	SHA. Lt	5.59	415	191	31	Yes, Larger (BG, Lt)	BG, Lt	3	8
3	SHA, Rt	6.4	409	113	56	Yes, Larger (BG Rt)	BG, Rt	22	9
4	ACoA, Lt	5.3	386	137	27	No	BG, Lt	137	26
5	ACoA, Lt	5.03	493	159	25	Yes, Larger (Frontal, Lt)	Frontal, Lt	5	10
6	BT, Lt	4.5	475	225	19	Yes, Larger (Cbll, Lt)	Cbll, Lt	2	13
7	ICA dorsal, Lt	4.1	483	70	68	Yes, Larger (Insula, Lt)	Insula, Lt	2	2
8	SHA Rt	4.3	412	153	42	Yes, Larger (BG, Rt)	BG, Rt	4	15

*ACoA, anterior communicating artery; BG, basal ganglia; BT, basilar top; Cbll, cerebellar; CN, caudate nucleus; ICA, internal carotid artery; Lt, left; Rt, right; SHA, superior hypophyseal artery*.

### Results of Multivariate Analysis

Multivariate analysis revealed two significant risk factors for post-procedural d-ICH (Table 5). The larger post DWI positivity (OR = 25.3, 95% CI = 5.763–111.565, *P* = 0.001) and the value of PRU (OR = 0.039, 95% CI = 0.970–0.999, *P* = 0.039) were those risk factors. Stent deployment did not reach statistical significance by this analysis.

**Table 5:** Multivariate analysis for risk factors of post-procedural ICH.

	***p*-value**	**OR**	**95% CI**
Larger DWI positive lesion	0.001	83.73	11.132–712.886
PRU	0.033	0.98	0.972–0.999

## Discussion

### Overall Results of Our Study

This study analyzed DWI and DAPT for risk factors of d-ICH after coil embolization for an UIA. Although there are several studies on d-ICH after endovascular treatment of an UIA, risk factor analysis about d-ICH specifically on coil embolization has not been sufficiently investigated. The major findings of this study were as follows: (1) the total incidence of d-ICH was 1.5%, with lesions identified on average 11.8 days after the procedure; (2) in most of our cases, d-ICH was identified exactly at the larger DWI positive lesion on 1-day follow-up DWI MRI; (3) larger DWI lesion, lower PRU, and higher inhibition rate were significant risk factors for d-ICH in univariate analysis. In multivariate analysis, larger DWI lesion and PRU value showed statistical significance. These results may indicate that a tailored reduction in antiplatelet agents can help reduce d-ICH when a larger volume of post-procedural thromboembolic infarction is seen on 1-day follow-up DWI MRI.

### Relationship Between d-ICH and DWI Positive Lesions

In most of our cases, larger post-procedural DWI positive lesions changed into d-ICH lesions, which means hemorrhagic transformation. We summarized clinical data and images of d-ICH patients (Table 4, Figure 1) and found that seven of eight cases of d-ICH were identified in the exact same areas of post-procedural infarction on day-1 DWI MRI (Case No 1, 2, 3, 5, 6, 7, and 8). Accordingly, it seems important to perform 1-day follow-up DWI MRI after the procedure. However, case No. 4 was somewhat exceptional compared to the other patients. He had hypertension and a previous history of antiplatelet medication. His laboratory values of ARU, PRU, and inhibition rate were not extreme. He underwent stent-assisted coil embolization successfully, and no definite procedure-related complications occurred. His day-1 follow-up DWI showed no positive ischemic lesions, and even his follow-up CT scan was unremarkable. However, on his 26th day after the procedure, he had a massive ICH on the left basal ganglia and expired during ICU care after evacuation surgery. We suppose the etiology of his ICH might differ from others, which might be related to senility or hypertensive microangiopathy.

Of interest, small tiny embolic infarctions might have less chance of hemorrhagic transformation. According to our subgroup analysis of DWI lesions (Table 2), seven of eight patients in the d-ICH positive group had a large DWI lesion without a microembolic lesion. Therefore, despite the small number of cases, we carefully hypothesized that a relatively large-size infarcted lesion (more than area 10 mm^2^) was more likely to change into a d-ICH.

There have been a few studies on hemorrhagic transformation after endovascular procedures for UIA. Brinjikji et al. showed that ICH incidence increased 4-fold after treatment with multiple flow diverters due to secondary hemorrhagic transformation of the resulting ischemic infarcts (11). Nakae et al. hypothesized that hemorrhagic transformation of a post-procedural ischemic lesion may be the main cause of d-ICH following flow diverter treatment. Furthermore, flow diverter-treated patients might have an increased risk of hemorrhagic transformation compared with those treated with standard stent-assisted coiling methods (16). However, these studies only have dealt with flow diverters. We cautiously suggest that hemorrhagic transformation may be an important risk factor not only in flow diverter treatment but also in coiling embolization, especially in stent-assisted methods.

### Relationship Between d-ICH and Antiplatelet Medication

The possibility that a higher response to the antiplatelet agents might impact d-ICH risk is another consideration. A few studies have reported on the relationship between post-procedural ICH and antiplatelet medication. Shapiro et al. reported that the hemorrhagic risk of stent-assisted coil embolization of the UIA with DAPT is 2.2–2.6% (18, 19). Zhang et al. reported that delayed hemorrhage was associated with the use of antiplatelet agents before the procedure (4). Goh et al. reported that a hyper-response to the antiplatelet effects of clopidogrel is associated with a significant increase in bleeding risk in the setting of endovascular neurointervention (3). Our study is partially concordant with these previous reports. Especially, Sim et al. demonstrated that four mechanisms have been discussed as potential causes of ICH after endovascular treatment. These mechanisms are DAPT, hemorrhagic transformation of clinically silent small periprocedural embolic infarcts, intraprocedural foreign body emboli, and flow modification (8). They considered these mechanisms as individually independent factors.

We sought to add some ideas about those reports. Our multivariate study showed that two possible risk factors have meaningful significance. We hypothesized that d-ICH might not occur only due to a single factor. Several factors could be considered together as risk factors, and they might affect each other. For example, DWI positive lesions and higher response to antiplatelet agents could both contribute as factors. Admittedly, our study is not large enough to confirm tight relations between these two factors. However, we thought that DWI positivity is one of the most important factors, and antiplatelet responsiveness also cannot be dismissed. And also, despite less statistical significance, stent deployment and DAPT should not be dismissed as potential risk factors for d-ICH, because all patients with d-ICH underwent stent deployment and were given DAPT. We thought that the poor statistical value of these factors might be because of the small number of d-ICH patients. As a result, we can suggest that if a larger DWI positive lesion is found in 1-day follow-up DWI MRI, a tailored dose reduction of the antiplatelet agent or perhaps a change from DAPT to SAPT may be beneficial to prevent d-ICH.

There are several limitations to our study. First, this is a non-randomized, single-center retrospective study. So the results might be influenced by the limitations of the study design. Second, we were only able to perform ARU and PRU labs once at the beginning of the procedure. Future studies on this should consider follow up laboratory studies on ARU and PRU labs with multiple measurements. Third, the cohort size was modest, especially given the small number of d-ICH patients. Further large scale studies are needed to confirm our hypothesis.

## Conclusion

d-ICH after coil embolization of an UIA may be linked to hemorrhagic transformation of a thromboembolic infarction and higher response to antiplatelet medications. Based on our data, we cautiously suggest that a tailored reduction in antiplatelet agents could be helpful to reduce d-ICH when a large volume of post-procedural infarction is seen on 1-day DWI MRI after coiling of UIA. Further studies are needed to strengthen these preliminary findings.

## Data Availability Statement

The original contributions presented in the study are included in the article/supplementary materials, further inquiries can be directed to the corresponding author/s.

## Ethics Statement

The studies involving human participants were reviewed and approved by the institutional review board at Kyoungpook National University Hospital. Due to the retrospective nature of the study, written informed consent was not required.

## Author Contributions

D-HK contributed to the preparation of the manuscript, data analysis, interpretation, and revision of the manuscript. WS contributed to data collection and writing of the manuscript. All authors contributed to the article and approved the submitted version.

## Conflict of Interest

The authors declare that the research was conducted in the absence of any commercial or financial relationships that could be construed as a potential conflict of interest.

## References

[1] Bhatt DL Kapadia SR Bajzer CT Chew DP Ziada KM Mukherjee D. Dual antiplatelet therapy with clopidogrel and aspirin after carotid artery stenting. J Invasive Cardiol. (2001) 13:767–71. 11731685

[2] Bracard S Barbier C Derelle AL Anxionnat R. Endovascular treatment of aneurisms: pre, intra and post operative management. Eur J Radiol. (2013) 82:1633–7. 10.1016/j.ejrad.2013.02.012 23478007

[3] Goh C Churilov L Mitchell P Dowling R Yan B. Clopidogrel hyper-response and bleeding risk in neurointerventional procedures. AJNR Am J Neuroradiol. (2013) 34:721–6. 10.3174/ajnr.A3418 23275598 PMC7964475

[4] Zhang XD Wu HT Zhu J He ZH Chai WN Sun XC. Delayed intracranial hemorrhage associated with antiplatelet therapy in stent-assisted coil embolized cerebral aneurysms. Acta Neurochir Suppl. (2011) 110 (Pt 2):133–9. 10.1007/978-3-7091-0356-2_24 21125459

[5] Cuisset T Frere C Quilici J Gaborit B Castelli C Poyet R. Predictive values of post-treatment adenosine diphosphate-induced aggregation and vasodilator-stimulated phosphoprotein index for stent thrombosis after acute coronary syndrome in clopidogrel-treated patients. Am J Cardiol. (2009) 104:1078–82. 10.1016/j.amjcard.2009.06.007 19801028

[6] Delgado Almandoz JE Crandall BM Scholz JM Fease JL Anderson RE Kadkhodayan Y. Pre-procedure P2Y12 reaction units value predicts perioperative thromboembolic and hemorrhagic complications in patients with cerebral aneurysms treated with the pipeline embolization device. J Neurointerv Surg. (2013) 5 (Suppl. 3):iii3–10. 10.1136/neurintsurg-2012-010582 23314576

[7] Sibbing D Schulz S Braun S Morath T Stegherr J Mehilli J. Antiplatelet effects of clopidogrel and bleeding in patients undergoing coronary stent placement. J Thromb Haemost. (2010) 8:250–6. 10.1111/j.1538-7836.2009.03709.x 19943882

[8] Sim SY Song J Oh SY Kim MJ Lim YC Park SK. Incidence and characteristics of remote intracerebral hemorrhage after endovascular treatment of unruptured intracranial aneurysms. World Neurosurg. (2016) 95:335–40. 10.1016/j.wneu.2016.08.057 27565469

[9] Baldi G Altomonte F Altomonte M Ghirarduzzi A Brusasco C Parodi RC. Intracranial haemorrhage in patients on antithrombotics: clinical presentation and determinants of outcome in a prospective multicentric study in Italian emergency departments. Cerebrovasc Dis. (2006) 22:286–93. 10.1159/000094604 16847397

[10] Delgado Almandoz JE Kadkhodayan Y Crandall BM Scholz JM Fease JL Tubman DE. Variability in initial response to standard clopidogrel therapy, delayed conversion to clopidogrel hyper-response, and associated thromboembolic and hemorrhagic complications in patients undergoing endovascular treatment of unruptured cerebral aneurysms. J Neurointerv Surg. (2014) 6:767–73. 10.1136/neurintsurg-2013-010976 24353331

[11] Brinjikji W Lanzino G Cloft HJ Siddiqui AH Kallmes DF. Risk factors for hemorrhagic complications following pipeline embolization device treatment of intracranial aneurysms: results from the international retrospective study of the pipeline embolization device. AJNR Am J Neuroradiol. (2015) 36:2308–13. 10.3174/ajnr.A4443 26251427 PMC7964264

[12] Chalouhi N Zanaty M Jabbour PM Starke RM Tjoumakaris SI Rosenwasser RH. Intracerebral hemorrhage after pipeline embolization: management of antiplatelet agents and the case for point-of-care testing–case reports and review of literature. Clin Neurol Neurosurg. (2014) 124:21–4. 10.1016/j.clineuro.2014.06.021 24999277

[13] Cruz JP Chow M O'Kelly C Marotta B Spears J Montanera W. Delayed ipsilateral parenchymal hemorrhage following flow diversion for the treatment of anterior circulation aneurysms. AJNR Am J Neuroradiol. (2012) 33:603–8. 10.3174/ajnr.A3065 22403783 PMC8050437

[14] Fargen KM Hoh BL. Ipsilateral cerebral hemorrhage following deployment of the pipeline embolization device. J Neurosurg. (2014) 120:363–4. 10.3171/2013.10.JNS132111 24320026

[15] Hu YC Deshmukh VR Albuquerque FC Fiorella D Nixon RR Heck DV. Histopathological assessment of fatal ipsilateral intraparenchymal hemorrhages after the treatment of supraclinoid aneurysms with the pipeline embolization device. J Neurosurg. (2014) 120:365–74. 10.3171/2013.11.JNS131599 24320006

[16] Nakae R Nagaishi M Kawamura Y Tanaka Y Hyodo A Suzuki K Microhemorrhagic transformation of ischemic lesions on T2^*^-weighted magnetic resonance imaging after pipeline embolization device treatment. J Neurosurg. (2018) 1:1–8. 10.3171/2017.12.JNS172480 29999443

[17] Velat GJ Fargen KM Lawson MF Hoh BL Fiorella D Mocco J. Delayed intraparenchymal hemorrhage following pipeline embolization device treatment for a giant recanalized ophthalmic aneurysm. J Neurointerv Surg. (2012) 4:e24. 10.1136/neurintsurg-2011-010129 21990545

[18] King B Vaziri S Singla A Fargen KM Mocco J. Clinical and angiographic outcomes after stent-assisted coiling of cerebral aneurysms with enterprise and neuroform stents: a comparative analysis of the literature. J Neurointerv Surg. (2015) 7:905–9. 10.1136/neurintsurg-2014-011457 25352581

[19] Shapiro M Becske T Sahlein D Babb J Nelson PK. Stent-supported aneurysm coiling: a literature survey of treatment and follow-up. AJNR Am J Neuroradiol. (2012) 33:159–63. 10.3174/ajnr.A2719 22033717 PMC7966171

